# 单纯输注抗BCMA与抗BCMA输注联合抗CD19嵌合抗原受体T细胞治疗复发/难治性多发性骨髓瘤免疫重建的对比

**DOI:** 10.3760/cma.j.issn.0253-2727.2021.09.004

**Published:** 2021-09

**Authors:** 娇 葛, 婷婷 赵, 重阳 万, 洁云 夏, 思邑 郭, 明潇 于, 娟 陈, 莹 王, 开林 徐, 振宇 李

**Affiliations:** 徐州医科大学附属医院血液科 221002 Department of Hematology, the Affiliated Hospital of Xuzhou Medical University, Xuzhou 221002, China

**Keywords:** 嵌合抗原受体T细胞, 多发性骨髓瘤, 免疫重建, Chimeric antigen receptor T cells, Multiple myeloma, Immune reconstruction

## Abstract

**目的:**

观察比较单纯输注抗B细胞成熟抗原（BCMA）嵌合抗原受体T细胞（CAR-T细胞）与输注抗BCMA联合抗CD19 CAR-T细胞治疗复发/难治性多发性骨髓瘤（RRMM）患者免疫重建的差异。

**方法:**

以2017年6月至2020年12月在我院行CAR-T细胞治疗的61例RRMM患者为研究对象，其中单纯接受抗BCMA靶点者26例，接受抗BCMA联合抗CD19靶点者35例。流式细胞术分别测定CAR-T治疗前后不同时间点的T细胞亚群（CD3^+^、CD4^+^、CD8^+^、CD4^+^/CD8^+^）、B细胞（CD19^+^）及NK细胞（CD16^+^CD56^+^），免疫比浊法检测免疫球蛋白IgG、IgA及IgM水平。对比两组淋巴细胞亚群及免疫球蛋白的重建规律。

**结果:**

CAR-T细胞输注后，CD8^+^T淋巴细胞恢复最快，BCMA组与BCMA+CD19组分别在输注后3个月和输注后1个月时恢复至输注前水平［BCMA：695（357, 1264）个/µl对424（280, 646）个/µl；BCMA+CD19：546（279, 1672）个/µl对314（214, 466）个/µl］。两组NK细胞均在输注后3个月时恢复至正常水平［分别为171（120, 244）个/µl、153（101, 218）个/µl（正常参考范围150～1100个/µl）］，但BCMA组不能维持在稳定水平。两组CD4^+^ T淋巴细胞恢复缓慢，输注后12个月内仍处于持续低水平，多数患者未观察到恢复。CD4^+^/CD8^+^比值倒置持续1年以上。两组CD19^+^B细胞均在输注后3个月恢复至输注前水平［BCMA：62（10, 72）个/µl对57（24, 78）个/µl；BCMA+CD19：40（4, 94）个/µl对29（14, 46）个/µl］。BCMA组IgG在输注后12个月恢复至输注前水平［7.82（6.03, 9.64）g/L对6.92（4.62, 12.76）g/L］，BCMA+CD19组未恢复。IgA先后在输注后9个月和12个月恢复至输注前水平［BCMA：0.46（0.07, 0.51）g/L对0.22（0.12, 4.01）g/L；BCMA+CD19：0.46（0.22, 0.98）g/L对0.27（0.10, 0.53）g/L］。两组IgM在输注后6个月恢复至输注前水平［BCMA：0.43（0.06, 0.60）g/L对0.20（0.13, 0.37）g/L；BCMA+CD19：0.53（0.10, 0.80）g/L对0.16（0.11, 0.28）g/L］。两组患者淋巴细胞亚群重建及免疫球蛋白恢复在各时间点的差异均无统计学意义。

**结论:**

在接受CAR-T细胞治疗的RRMM患者中，可基本不考虑单纯抗BCMA CAR-T与抗BCMA联合抗CD19 CAR-T治疗之间免疫重建的差异而选择合适的靶抗原。**中国临床试验注册中心：**ChiCTR-OIC-17011271、ChiCTR-OIC-17011272

## Abstract (en)

**Objective:**

We observed and compared the differences in immune reconstruction between single-infusion anti-B-cell maturation antigen（BCMA）, chimeric antigen receptor T cells（CAR-T）, and combined infusion of anti-CD19 CAR-T cells in the treatment of recurrent/refractory multiple myeloma（RRMM）.

**Methods:**

Sixty-one patients with RRMM who underwent CAR-T cell therapy in our hospital from June 2017 to December 2020 were selected. Among them, 26 patients received anti-BCMA target, and 35 patients received anti-BCMA combined with anti-CD19 target. Using flow cytometry, we determined T cell subsets（CD3^+^, CD4^+^, CD8^+^, CD4^+^/CD8^+^）, B cells（CD19^+^）, and NK cells（CD16^+^ CD56^+^）at different time points before and after CAR-T treatment, and detected immunoglobulin IgG, IgA and IgM levels by immunoturbidimetry. We compared the reconstruction rules of lymphocyte subsets and immunoglobulins in the two groups.

**Results:**

CD8 ^+^ T lymphocytes recovered most rapidly after the infusion of CAR-T cells, returning to pre-infusion levels at 3 months and 1 month after infusion, respectively［BCMA: 695（357, 1264）/µl *vs* 424（280, 646）/µl; BCMA+CD19: 546（279, 1672）/µl *vs* 314 （214, 466）/µl］. NK cells returned to normal levels at 3 months after infusion in both groups［BCMA: 171 （120, 244）/µl, BCMA+CD19: 153（101, 218）/µl（Normal reference range 150–1100 /µl）］; however, the NK cells were not maintained at stable levels in the BCMA CAR-T cells group. The recovery of CD4^+^ T lymphocytes in both groups was slow and remained persistently low within 12 months after infusion, and no recovery was observed in most patients. The reversal of the ratio of CD4^+^/CD8^+^ lasted for more than a year. The levels of CD19^+^ B cells in both groups returned to baseline 3 months after infusion［BCMA: 62（10, 72）/µl *vs* 57（24, 78）/µl; BCMA+CD19: 40（4, 94）/µl *vs* 29（14, 46）/µl］. IgG returned to the pre-infusion level 12 months after infusion in the group with anti-BCMA cells alone, but not in the group with combined infusion of CD19 CAR T cells［7.82（6.03, 9.64）g/L *vs* 6.92（4.62, 12.76）g/L］. IgA returned to pre-infusion levels at 9 and 12 months after infusion, respectively［BCMA: 0.46（0.07, 0.51）g/L *vs* 0.22 （0.12, 4.01）g/L; BCMA+ CD19: 0.46（0.22, 0.98）g/L *vs* 0.27（0.10, 0.53）g/L］. IgM in both groups returned to pre-infusion levels 6 months after infusion［BCMA: 0.43（0.06, 0.60）g/L *vs* 0.20（0.13, 0.37）g/L; BCMA+CD19: 0.53（0.10, 0.80）g/L *vs* 0.16（0.11, 0.28）g/L］. There was no significant difference in the indexes of lymphocyte subpopulation reconstruction and immunoglobulin recovery between the two groups at each time point.

**Conclusion:**

This study showed that in patients with RRMM treated with CAR-T cells, the appropriate target antigen can be selected without considering the difference of immune reconstruction between anti-BCMA CAR-T and combined anti-CD19 CAR-T therapy.

多发性骨髓瘤（MM）是一种高度异质性疾病，其发病机制尚未完全阐明。在过去十年中，蛋白酶体抑制剂、免疫调节剂及造血干细胞移植等方法的应用使得MM的治疗效果得到了提高，患者生存期延长[1]–[2]，但复发难治仍使相当一部分患者难以获益[3]。嵌合抗原受体T细胞（CAR-T细胞）疗法作为一种特殊的肿瘤细胞免疫治疗方式，在复发难治B细胞肿瘤患者中显示出令人欣喜的临床疗效[4]–[6]。然而CAR-T治疗后的不良反应也不容忽视，例如细胞因子释放综合征（CRS）[7]–[8]和对正常B细胞的杀伤作用。先前已有研究报道了CAR-T细胞治疗后B细胞再生障碍和低免疫球蛋白血症[9]–[10]，但关于免疫系统恢复规律尚鲜有文献报道。本研究我们回顾性分析徐州医科大学附属医院血液科61例复发难治性MM（RRMM）患者资料，比较单纯输注抗B细胞成熟抗原（BCMA）CAR-T细胞与联合输注抗CD19 CAR-T细胞治疗后免疫重建的差异。

## 病例与方法

1. 病例资料：回顾性分析2017年6月至2020年12月于徐州医科大学附属医院血液科接受CAR-T治疗并且临床相关资料完整的RRMM患者共61例。其中单纯输注抗BCMA CAR-T细胞26例，男17例、女9例，中位年龄为56（52～63）岁，按M蛋白亚型分为IgG型5例、IgA型8例、轻链型11例、IgD型2例；输注抗BCMA联合抗CD19 CAR-T细胞组35例，男21例、女14例，中位年龄为59（45～66）岁，按M蛋白亚型分为IgG型18例、IgA型7例、轻链型6例、不分泌型2例、IgD型2例。纳入标准：①符合《中国多发性骨髓瘤诊治指南（2020年修订）》[11]中RRMM标准；②18～69岁；③ Karnofsky功能状态评分≥50分；④预期寿命超过12周且无活动性感染、严重心肝肾功能损害、慢性消耗性疾病及其他影响生存的疾病；⑤入组前100 d内无自体造血干细胞移植史；⑥充分了解本研究且签署知情同意书，依从性较好，CAR-T细胞输注后继续住院观察，出院后定期门诊或住院复诊（总观察时间≥60 d）。所有患者均入组CAR-T细胞临床试验（中国临床试验注册中心：ChiCTR-OIC-17011271、ChiCTR-OIC-17011272），本研究获得徐州医科大学附属医院临床试验伦理委员会批准（批件号：XYFY2017-KL014-01、XYFY2017-KL013-01）。

2. 标本采集、淋巴细胞亚群及免疫球蛋白检测方法：分别在输注前第5天、输注当天、输注后1、3、6、9和12个月采集患者外周血。通过流式细胞仪检测包括CD3、CD4、CD8、CD19、CD16/CD56在内的淋巴细胞标志物。进行免疫表型分析时，为减少收集后的选择性损失，标本不经进一步分离即进行染色。用抗CD45-BV421、抗CD3-FITC、CD4-APC、CD8-Per-CP、CD19-PE-Cy7、CD16-PE和CD56-PE的单克隆抗体进行染色。T细胞亚群（CD4 ^+^、CD8^+^）、NK细胞（CD16^+^/CD56^+^）和B细胞（CD19^+^）通过单一平台检测。合适的同型对照包括PE、APC和Per-CP偶联的IgG1/2a/2b。结合血常规检查所示外周血中淋巴细胞绝对数，分别计算其绝对数。采用免疫比浊法检测血清IgG、IgA和IgM水平。

3. CAR-T输注前预处理方案：全部61例患者在CAR-T细胞输注前均采取FC（氟达拉滨+环磷酰胺）方案进行预处理：氟达拉滨30 mg·m^−2^ ·d^−1^，−5～−3 d；环磷酰胺750 mg/m，−5 d。

4. CAR-T的制备及输注：于−14 d采用COMTEC型血细胞单采仪［费森尤斯卡比（中国）投资有限公司］单采患者外周血淋巴细胞，进行抗BCMA和抗CD19 CAR-T细胞制备。抗CD19 CAR-T细胞由抗CD19抗体scFv、CD8铰链区、4-1BB共刺激分子及CD3ζT细胞活化序列组成；抗BCMA CAR-T细胞应用慢病毒转染技术，由抗BCMA抗体scFv、4-1BB共刺激分子及CD3ζT细胞活化序列构成。患者CAR-T细胞回输剂量均为1×10^6^/kg。

5. 统计学处理：本研究数据均采用SPSS 24.0进行统计学处理。计量资料以中位数（第一四分位数，第三四分位数）描述，组间比较采用Kruskal-Wallis *H*检验或Mann-Whitney *U*检验，以*P*<0.05为差异有统计学意义。

## 结果

一、淋巴细胞亚群恢复情况（表1）

单纯输注抗BCMA CAR-T细胞后，T细胞重建规律：CD3^+^、CD8^+^ T细胞计数在输注当天均显著低于输注前水平，CD3^+^ T细胞计数输注后3个月恢复至输注前水平，CD8^+^ T细胞计数在输注后3个月恢复至输注前水平并达正常水平；CD4^+^ T细胞计数在输注当天及输注后3个月内显著低于输注前水平，输注后恢复缓慢，12个月内未达输注前水平；CD4^+^/CD8^+^比值在输注当天短暂升高，在输注后显著下降，比值倒置持续了至少1年。B细胞重建规律：在输注当天及输注后1个月显著低于输注前水平，输注后3个月恢复至输注前水平。NK细胞重建规律：在输注当天及输注后1个月显著低于输注前水平，在输注后3个月恢复至正常水平，在12个月内未恢复至输注前水平。

**表1:** 单纯输注抗BCMA CAR-T细胞组与输注抗BCMA联合抗CD19 CAR-T细胞组治疗前后不同时间点淋巴细胞亚群计数及比值变化［*M*（*P25, P75*）

时间	CD3^+^ T细胞计数（个/µl）	CD4^+^ T细胞计数（个/µl）	CD8^+^ T细胞计数（个/µl）	CD4^+^/CD8^+^比值	CD19^+^B细胞计数（个/µl）	NK细胞计数（个/µl）
单纯输注抗BCMA						
输注前	728（552,1119）	313（228,442）	424（280,646）	0.67（0.48,0.94）	57（24,78）	221（147,300）
输注当天	89（14,203）^a^	50（7,93）^a^	32（4,76）^a^	1.56（0.82,2.43）^c^	1.8（0,3）^a^	11（2,24）^a^
输注后1个月	680（413,1079）	189（117,313）^a^	294（238,680）	0.51（0.28,0.78）	0（0,1）^a^	72（45,156）^a^
输注后3个月	979（533,1788）	177（125,298）^a^	695（357,1264）	0.26（0.20,0.42）^a^	62（10,72）^b^	171（120,244）^b^
输注后6个月	1050（806,1268）	260（220,347）	672（510,949）^b^	0.39（0.26,0.59）^a^	146（13,214）^b^	144（90,225）
输注后9个月	966（522,1403）	208（174,326）	460（310,986）	0.43（0.37,0.61）	163（100,405）^b^	90（63,308）
输注后12个月	1107（867,1325）	304（230,404）	635（421,847）	0.42（0.30,0.61）	182（131,339）^b^	124（112,242）
输注抗BCMA联合抗CD19						
输注前	676（485,972）	294（216,465）	314（214,466）	0.98（0.64,1.69）	29（14,46）	213（111,324）
输注当天	90（77,180）^a^	57（41,82）^a^	30（19,63）^a^	1.78（0.88,3.20）	2（0,4）^a^	12（5,29）^a^
输注后1个月	793（528,1975）	217（113,388）	546（279,1672）^a^	0.28（0.17,0.61）^a^	0（0,0）^a^	101（44,182）^a^
输注后3个月	1078（771,1491）	240（180,358）	770（420,1021）^a^	0.35（0.29,0.56）^a^	40（4,94）^b^	153（101,218）
输注后6个月	1085（645,1258）	230（185,307）	661（378,872）^a^	0.37（0.26,0.58）^a^	111（58,171）^b^	189（110,268）
输注后9个月	952（610,1097）	298（165,402）	518（264,775）	0.58（0.43,0.84）	159（97,260）^a^	166（120,183）
输注后12个月	967（594,1363）	265（163,275）	675（243,990）	0.36（0.27,0.79）	163（97,194）^a^	164（92,182）

正常参考范围	955～2860	550～1440	320～1250	0.9～3.6	90～560	150～1100

注：BCMA：B细胞成熟抗原；CAR-T细胞：嵌合抗原受体T细胞。与输注前相比，^a^*P*<0.05；与输注后1个月相比，^b^*P*<0.05；与输注后1～6个月相比，^c^*P*<0.05

输注抗BCMA联合抗CD19 CAR-T细胞后，T细胞重建规律：CD3^+^、CD4^+^、CD8^+^ T细胞计数在输注当天均显著低于输注前水平，CD3^+^ T细胞计数输注后1个月恢复至输注前水平。CD4^+^ T细胞计数输注后恢复缓慢，12个月内未达输注前水平。CD8^+^ T细胞计数在输注后1个月显著高于输注前水平。CD4^+^/CD8^+^比值在输注当天短暂升高，在输注后6个月内显著低于输注前水平，比值倒置持续了至少1年。B细胞重建规律：在输注当天及输注后1个月显著低于输注前水平，输注后3个月恢复至输注前水平，在输注9个月后明显高于输注前水平。NK细胞重建规律：在输注当天及输注后1个月显著低于输注前水平，在输注后3个月恢复至正常水平，但在12个月内未恢复至输注前水平。

比较两组各时间点淋巴细胞亚群计数及CD4^+^/CD8^+^比值差异均无统计学意义（*P*值均>0.05）。联合输注抗CD19 CAR-T细胞组CD8^+^ T淋巴细胞恢复正常水平所需的时间较单输抗BCMA CAR-T细胞组稍长。虽两组NK细胞均在输注后3个月时恢复正常水平，但单纯输注抗BCMA CAR-T细胞组不能维持在稳定水平。

二、免疫球蛋白恢复情况（表2）

单纯输注抗BCMA CAR-T细胞后，IgG在输注后3个月时水平最低，3～6个月显著低于输注前水平，在输注后12个月恢复至输注前水平。IgA在输注后1～3个月显著低于输注前水平，在输注后9个月恢复至输注前水平。IgM在输注后1个月显著低于输注前水平，输注后6个月恢复至输注前水平，输注后12个月恢复至正常水平。

**表2:** 单纯输注抗BCMA CAR-T细胞组与输注抗BCMA联合抗CD19 CAR-T细胞组治疗前后不同时间点免疫球蛋白变化［g/L，*M*（*P25, P75*）］

时间	IgG	IgA	IgM
单纯输注抗BCMA			
输注前	6.92（4.62,12.76）	0.22（0.12,4.01）	0.20（0.13,0.37）
输注后1个月	4.58（3.20,11.10）	0.07（0.07,0.55）^a^	0.10（0.07,0.13）^a^
输注后3个月	3.59（1.83,7.43）^a^	0.07（0.07,0.09）^a^	0.10（0.05,0.32）
输注后6个月	4.33（0.98,6.28）^a^	0.14（0.07,0.37）	0.43（0.06,0.60）^b^
输注后9个月	5.57（2.66,6.94）	0.46（0.07,0.51）	0.44（0.18,0.67）^b^
输注后12个月	7.82（6.03,9.64）	0.72（0.53,1.14）^d^	0.97（0.39,1.19）^c^
输注抗BCMA联合抗CD19			
输注前	8.54（6.20,37.40）	0.27（0.10,0.53）	0.16（0.11,0.28）
输注后1个月	6.70（3.92,16.80）	0.07（0.07,0.22）^a^	0.12（0.06,0.16）
输注后3个月	4.36（3.70,9.20）^a^	0.07（0.07,0.20）^a^	0.12（0.05,0.33）
输注后6个月	5.35（2.47,8.12）^a^	0.10（0.07,0.37）	0.53（0.10,0.80）^c^
输注后9个月	5.53（4.70,7.53）	0.23（0.14,1.18）	0.47（0.33,1.03）^a^
输注后12个月	6.20（4.51,9.80）	0.46（0.22,0.98）^c^	0.54（0.27,0.83）^c^

正常参考范围	8.00～17.00	1.00～4.90	0.50～3.20

注：BCMA：B细胞成熟抗原；CAR-T细胞：嵌合抗原受体T细胞。与输注前相比，^a^*P*<0.05；与输注后1个月相比，^b^*P*<0.05；与输注后1～3个月相比，^c^*P*<0.05；与输注后1～6个月相比，^d^*P*<0.05

输注抗BCMA联合抗CD19 CAR-T细胞后，IgG在输注后3个月时水平最低，3～6个月显著低于输注前水平，后恢复较慢，在观察期内未达输注前水平。IgA在输注后1～3个月显著低于输注前水平，在输注后12个月恢复至输注前水平。IgM在输注后低于输注前水平，但差异无统计学意义，在输注后9个月显著高于输注前水平，输注后12个月达正常水平。

比较单纯输注抗BCMA CAR-T细胞组与输注抗BCMA联合抗CD19 CAR-T细胞组免疫球蛋白水平差异。CAR-T细胞输注后，所有患者均出现免疫球蛋白浓度下降和低免疫球蛋白血症，对比两组各时间点免疫球蛋白水平差异均无统计学意义（*P*值均>0.05）。输注抗BCMA联合抗CD19 CAR-T细胞组IgG、IgA恢复至输注前水平所需的时间均较单纯输注抗BCMA CAR-T细胞组稍长。虽差异无统计学意义，但在输注后12个月时，单纯输注抗BCMA CAR-T细胞组各类免疫球蛋白水平中位数均比输注抗BCMA联合抗CD19 CAR-T细胞组高。

## 讨论

“B细胞再生障碍”是CAR-T细胞疗法的一个主要毒性。BCMA在正常和恶性浆细胞中均高表达，在其他细胞谱系中几乎不存在[12]–[13]，也因此健康浆细胞可能受到CAR-T细胞的影响而引起继发性低免疫球蛋白血症。同样，抗CD19 CAR-T细胞疗法可通过耗竭CD19^+^B祖细胞而导致B细胞再生障碍[14]。在一项单中心1-2a期研究中[15]，所有输注抗CD19 CAR-T细胞且对治疗有反应的患者均发生了B细胞再生障碍，持续至6个月的概率为83％（95％ *CI* 69％～91％）。相比之下，关于比较单纯输注抗BCMA CAR-T细胞与输注抗BCMA联合抗CD19 CAR-T细胞治疗后免疫重建的详细报道很少。

淋巴细胞清除化疗后，患者淋巴细胞亚群的绝对计数显著下降，可能与FC方案进行的淋巴细胞清除化疗引起的淋巴细胞计数降低有关。CD8^+^ T淋巴细胞恢复最快，两组分别在输注后3个月和1个月时恢复至输注前水平，两组NK细胞均在输注后3个月时恢复至正常水平，但单纯输注抗BCMA CAR-T细胞组不能维持在稳定水平，两组CD4^+^ T淋巴细胞恢复缓慢，多数患者在输注后12个月内未观察到恢复。这可能与中老年患者胸腺的自然退化以及预处理时大剂量化疗导致胸腺功能受损有关，由于CD8^+^ T淋巴细胞生成和成熟不完全依赖胸腺而输注后较快恢复,但CD4^+^ T淋巴细胞的生成主要依赖于胸腺功能而恢复缓慢[16]，这也部分解释了输注后患者在较长时期内CD4^+^/CD8^+^比值下降及倒置的原因。CD4^+^/CD8^+^比值降低表明免疫受到抑制，是监测免疫恢复的重要标志[17]–[18]。输注CAR-T细胞后，CD4^+^/CD8^+^比值倒置持续了1年以上，这表明在CAR-T细胞输注后机体免疫功能失调持续了很长一段时间。与预处理前相比，CD19^+^ B淋巴细胞计数在输注后进一步降低并持续处于耗竭状态，是由淋巴细胞清除化疗引起淋巴细胞计数降低和CAR-T细胞的靶向杀伤作用导致。不同的淋巴细胞亚群有不同的功能，只有达到相互平衡与相互制约的状态，才能更好地发挥抗感染和抗肿瘤的作用。本研究中我们发现，对比单纯输注抗BCMA CAR-T细胞组与输注抗BCMA联合CD19 CAR-T细胞组在治疗前后各时间点淋巴细胞亚群计数及CD4^+^/CD8^+^比值差异均无统计学意义，说明两组在淋巴细胞亚群数量重建方面在同一水平。

本研究中，两组患者在输注CAR-T细胞后均发生持续性低免疫球蛋白血症。感染是降低骨髓瘤患者生命质量、影响疾病预后的重要因素[19]–[20]。CAR-T细胞输注后有两个主要的感染因素。一个是淋巴细胞清除化疗时抑制骨髓造血使得中性粒细胞缺乏，另一个是CAR-T细胞输注后引起的T细胞亚群异常、B细胞再生障碍及体液免疫受损有关。通常，骨髓抑制的恢复很快。然而，免疫功能的重建需要很长时间。

IgG是人免疫球蛋白的主要成分，也是初级免疫应答中最持久、最重要的抗体，在体液免疫中起着最重要的作用，是机体抗感染的“主力军”。本研究显示两组IgG在输注后6个月内持续下降，12个月时单纯输注抗BCMA CAR-T细胞组恢复输注前水平，考虑这可能与本研究中IgG型患者肿瘤负荷重有关，M蛋白的分泌抑制正常多克隆免疫球蛋白的合成，CAR-T细胞输注后，随着缓解程度的加深和M蛋白的代谢，在6个月后随着IgG水平的恢复，其体液免疫功能逐渐恢复。因此我们建议在输注CAR-T细胞后早期阶段反复感染又合并低免疫球蛋白的患者，应定期输注人免疫球蛋白。IgA中分泌型IgA是参与黏膜局部免疫的主要抗体类别，通过阻止病原微生物黏附到细胞表面在局部发挥抗感染作用，是机体抗感染的“边防军”，IgA低下易患呼吸道、胃肠道感染，本研究中，两组IgA先后在输注后9个月和12个月恢复输注前水平。IgM在机体早期免疫防护中占重要地位，主要存在于血管中，对防止血流感染具有重要意义，同时也是黏膜免疫的重要防线[21]，本研究中两组IgM在输注后6个月恢复输注前水平。

免疫球蛋白与各淋巴细胞亚群构成机体免疫防御机制的主要部分，其在抗肿瘤、免疫监视及抗感染中扮演重要角色。但数量重建只是免疫重建的一部分，而淋巴细胞功能的重建更为重要。本研究我们比较了单纯输注抗BCMA CAR-T细胞组与输注抗BCMA联合CD19 CAR-T细胞组在各时间点免疫重建指标差异均无统计学意义，这就意味着可在不考虑免疫重建的基础上选择合适的靶抗原，使RRMM患者最大程度获益。本研究因回顾性分析和样本量不足等局限性，如能进一步设计前瞻性随机对照研究可能能够获得更为明确的结论。
